# SERS-based aptasensor for culture-free detection of *Escherichia coli* in urinary tract infection diagnosis

**DOI:** 10.1186/s40580-025-00506-0

**Published:** 2025-08-21

**Authors:** Kihyun Kim, Sohyun Park, Suyoung Kang, Mi-Kyung Lee, Lingxin Chen, Jaebum Choo

**Affiliations:** 1 Department of Chemistry, Chung-Ang University, Seoul, 06974 South Korea; 2 Department of Laboratory Medicine, Chung-Ang University College of Medicine, Seoul, 06973 South Korea; 3 CAS Key Laboratory of Coastal Environmental Processes and Ecological Remediation, Yantai Institute of Coastal Zone Research, Yantai, 264003 China

**Keywords:** Surface-enhanced Raman scattering, Aptasensor, Urinary tract infection, *Escherichia coli*, Gold nanoparticle-embedded magnetic beads

## Abstract

A surface-enhanced Raman scattering (SERS)-based aptasensor was developed for the rapid and sensitive detection of *Escherichia coli* (*E. coli*), a major pathogen in urinary tract infections (UTIs). The sensor utilizes magnetic beads embedded with gold nanoparticles (MB-AuNPs) functionalized with capture DNA (cDNA) as both the SERS-active substrate and magnetic separation tool. The detection mechanism relies on an aptamer DNA-probe DNA complex: when the aptamer binds specifically to *E. coli*, the probe DNA is released and subsequently hybridizes with cDNA on the MB-AuNPs. This brings a Cy5 Raman label close to the gold surface, generating a strong SERS signal. The assay offers a one-step process, eliminating the need for bacterial culture or nucleic acid amplification, and completes within approximately 6 h. Quantitative analysis demonstrated a detection limit of 5.9 × 10^3^ CFU/mL, well below the clinical threshold for UTIs, with a reliable calibration curve (R^2^ = 0.990). Selectivity tests confirmed high specificity for *E. coli* without cross-reactivity to other bacteria. Clinical evaluation using 21 urine samples showed high diagnostic performance: 100% sensitivity, 91% specificity, 95% accuracy, and 100% precision compared to standard urine culture. These results highlight the aptasensor’s potential as a rapid, sensitive, and specific alternative for UTI diagnosis in clinical settings.

**Graphical Abstract:**

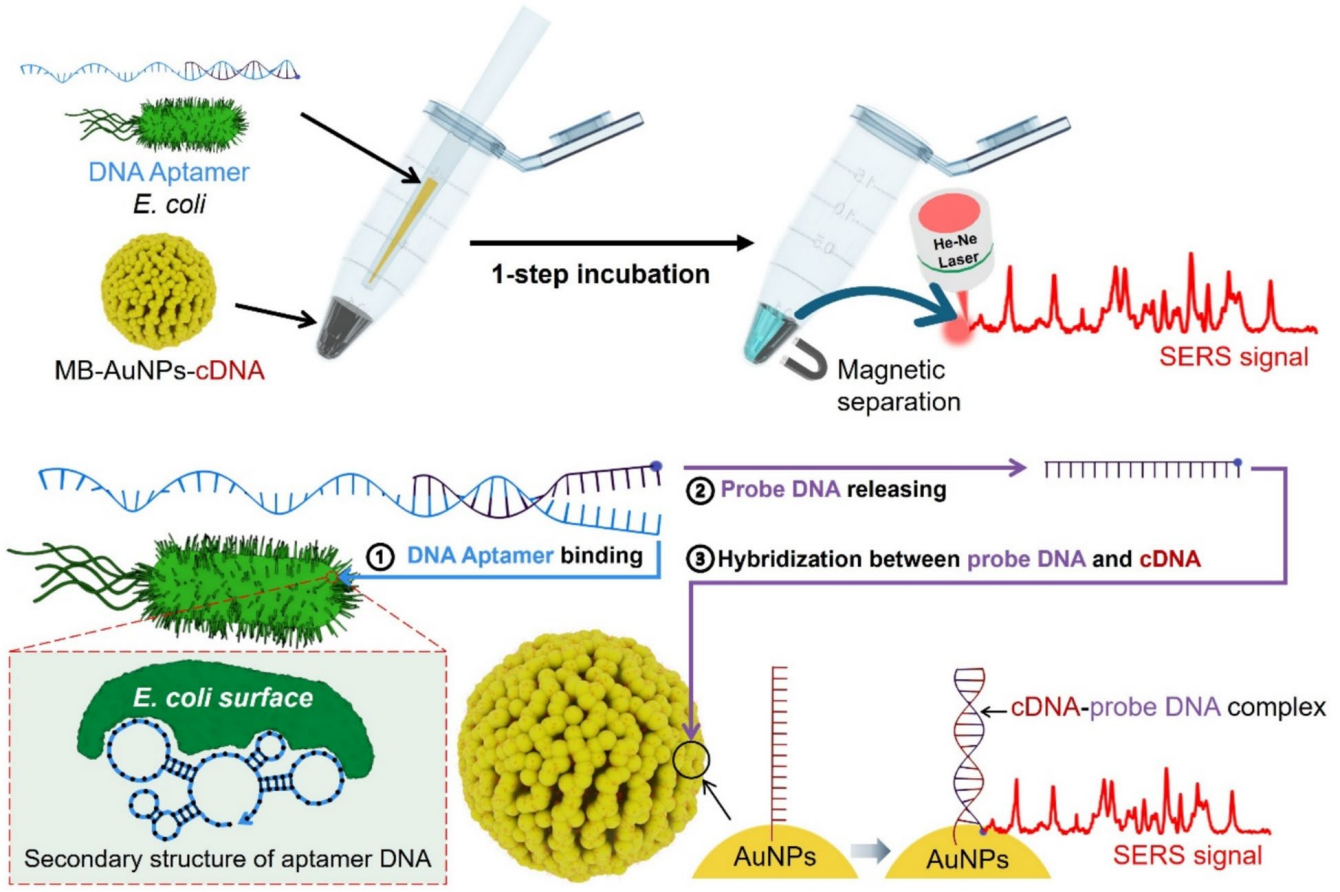

**Supplementary Information:**

The online version contains supplementary material available at 10.1186/s40580-025-00506-0.

## Introduction

Urinary tract infections (UTIs) are a significant global health concern, affecting millions of individuals annually [1]. These infections occur when microorganisms, primarily bacteria, invade the urinary system, including the kidneys, ureters, bladder, and prostate, triggering an inflammatory response [2–4]. While various bacterial species can cause UTIs, *Escherichia coli* (*E. coli*) is responsible for approximately 85% of cases, with other common pathogens including *Klebsiella pneumoniae*, *Proteus mirabilis*, *Enterococcus*, and *Staphylococcus saprophyticus* [5, 6]. The current diagnostic paradigm for UTIs relies on clinical evaluation based on urine culture tests. However, this traditional approach is time-consuming and labor-intensive, often requiring 48–72 h to deliver definitive results [7]. This delay frequently prompts clinicians to prescribe broad-spectrum antibiotics empirically before receiving test outcomes, contributing to antibiotic misuse and the growing problem of antimicrobial resistance. The primary reason for this delay lies in the time required for bacterial colony formation, which typically exceeds 24 h. Although molecular diagnostic techniques such as real-time polymerase chain reaction (RT-PCR) have emerged as promising alternatives by detecting target genes from lysed bacteria [8, 9], their widespread adoption is hindered by challenges such as complex preprocessing steps and reduced sensitivity and specificity due to interfering signals from urine components.

Surface-enhanced Raman scattering (SERS)-based bacterial assays have emerged as a rapid and sensitive method for pathogen detection, leveraging localized surface plasmon effects to enable low-concentration bacterial measurement with minimal cultivation [10, 11]. Despite advancements in high-sensitivity SERS sensing techniques [12–15], current approaches relying on antibody-conjugated magnetic beads (MBs) and SERS nanotags face two critical limitations that hinder clinical reliability. First, the mismatch between bacterial size (several micrometers) and Raman laser beam diameter (1–1.5 μm) causes inconsistent sampling, as only a fraction of bacteria enter the laser’s focal volume, leading to poor reproducibility due to variable SERS nanotag detection [16–19]. Second, conventional assays suffer from weak electromagnetic enhancement, as immunocomplexes formed between MBs and SERS nanotags lack nanogap structures, forcing reliance solely on nanotag-derived hot spots and resulting in subdued Raman signals that impede quantitative accuracy [12, 20, 21]. These challenges—rooted in measurement variability and insufficient signal amplification—severely limit the clinical adoption of SERS-based methods, where precise, reproducible quantification is essential for diagnosing UTIs and other bacterial diseases. Addressing these limitations is pivotal for advancing SERS into a robust diagnostic tool capable of meeting the demands of real-world clinical and public health applications.

This study presents a novel SERS-based aptasensor for detecting *E. coli*, a causative agent of UTIs, addressing critical limitations of poor reproducibility and weak electromagnetic enhancement effect in existing methods. Our approach employs double-stranded (ds) DNA—comprising an aptamer DNA with a strong binding affinity for bacterial surface proteins and a hybridized probe DNA—to selectively target *E. coli* [13, 22–25]. Upon bacterial binding, probe DNAs dissociate and hybridize with capture DNAs pre-immobilized on magnetic beads (MBs), enabling indirect Raman signal detection of the separated probes rather than direct measurement of bacterial complexes, thereby enhancing reproducibility. To amplify sensitivity, we replaced conventional bare MBs with AuNP-embedded MBs (MB-AuNPs), where 70 nm gold nanoparticles generate additional electromagnetic hot spots, significantly boosting signal intensity through localized surface plasmon resonance. This dual innovation—dsDNA-mediated indirect detection and MB-AuNPs-enhanced electromagnetic coupling—resulted in a robust, culture-free platform with high reproducibility and sensitivity. By resolving key bottlenecks in SERS-based assays, this aptasensor advances rapid, reliable *E. coli* detection, offering transformative potential for UTI diagnosis, antimicrobial stewardship, and food safety and environmental monitoring applications.

## Experimental section

### Reagents and materials

Gold (III) chloride trihydrate (HAuCl₄·3H₂O), trisodium citrate, 1-ethyl-3-(3-dimethylaminopropyl)carbodiimide (EDC), (S)-2-aminobutane-1,4-dithiol hydrochloride (DTBA), magnesium chloride, 6-mercapto-1-hexanol (6-MCH), tris(2-carboxyethyl)phosphine hydrochloride (TCEP), bovine serum albumin (BSA), and casein from bovine milk were obtained from Sigma-Aldrich (St. Louis, MO, USA). Phosphate-buffered saline (PBS) (10 × , pH 7.4), carboxylic acid-activated magnetic beads (Dynabeads MyOne^™^), and SYBR^™^ Safe DNA Gel Stain were obtained from Invitrogen (Eugene, OR, USA). Gel loading dye was purchased from New England Biolabs (Ipswich, MA, USA). 15% Mini-PROTEAN^®^ TBE Gel and 5% Mini-PROTEAN^®^ TBE Gel were purchased from Bio-Rad (Hercules, CA, USA). Luria–Bertani (LB) agar and broth were purchased from Genedepot (Katy, TX, USA). Ultrapure water (18.2 MΩ cm⁻^1^) used in this study was obtained from an arium^®^ comfort I system (Sartorius, Göttingen, Germany). *Escherichia coli* (*E. coli*) KCTC 2571 was obtained from the Korean Collection for Type Cultures (KCTC, Korea). *Staphylococcus aureus* (*S. aureus*) NCCP 14528 and methicillin-resistant *Staphylococcus aureus* (*MRSA*) NCCP 11489 were provided by the National Culture Collection for Pathogens (NCCP, Korea). The DNA sequences used in this study were obtained from Bioneer (Korea) and are detailed in Table 1.

**Table 1:** Base sequences of the aptamer DNA, capture DNA, complementary DNA, and non-complementary DNA used in this study

Name	Length (nt)	Sequence (5–3′)
Aptamer DNA	88	GCAATGGTACGGTACTTCCCCATGAGTGTTGTGAAATGTTGGGACACTAGGTGGCATAGAGCCGCAAAAGTGCACGCTACTTTGCTAA
Aptamer-Q	88	GCAATGGTACGGTACTTCCCCATGAGTGTTGTGAAATGTTGGGACACTAGGTGGCATAGAGCCGCAAAAGTGCACGCTACTTTGCTAA-BHQ2
Capture DNA (cDNA)	24	CAAAAGTGCACGCTACTTTGCTAA-(CH_2_)_3_-SH
Cy5-labeled cDNA (cDNA-Cy5)	24	CAAAAGTGCACGCTACTTTGCTAA-Cy5-(CH_2_)_3_-SH
Complementary DNA (probe DNA)	24	Cy5-TTAGCAAAGTAGCGTGCACTTTTG
Non-complementary DNA	20	Cy5-TAATATTCCTGTACTTGGTG

### Cultivation and concentration determination of *E. coli*

*E. coli* cultures were initially grown on LB agar plates at 37 °C for 24 h. A single colony was then isolated and inoculated into a conical tube containing 10 mL of LB broth, followed by incubation at 37 °C for 14 h under 200 rpm. Subsequently, 100 μL of the overnight culture was inoculated into 10 mL of fresh LB broth and sub-cultured at 37 °C for 3.5 h to obtain fresh cells in the exponential growth phase. The bacterial cells were washed three times with 1 × PBS (2000 g, 10 min) and the resulting cell suspension was aliquoted and stored at  - 70 °C until further use. *S. aureus* and *MRSA* were prepared following the same protocol described above. All reagents and glassware used in the experiments were sterilized by autoclaving at 121 °C for 15 min prior to use. A fresh bacterial cell suspension sub-cultured for 3.5 h was serially diluted in sterile 1 × PBS to prepare a range of dilutions (10⁻^1^ to 10⁻^5^). A 1 μL loop of each bacterial suspension was inoculated onto LB agar plates using a streaking method, followed by incubation at 37 °C for 24 h. Colonies were counted, and the colony forming unit (CFU)/mL of the bacterial stock solution was determined based on the dilution factor that resulted in colony counts within the range of 30–300.

### DNA hybridization between aptamer and probe DNAs

The aptamer DNAs specific to *E. coli* and the probe DNAs were selected and designed based on a previously reported paper [26–28]. A molar ratio of 1:1.5 of probe DNA to aptamer DNA was mixed in 1 × PBST buffer (0.05% v/v) and heated to 95 °C to denature the DNA strands. The mixture was then gradually cooled to 25 °C at a rate of - 1 °C/min to facilitate annealing. The resulting aptamer-probe dsDNAs were stored at 4 °C until further use. Successful hybridization between the aptamer and probe DNAs was confirmed by gel electrophoresis. This was performed by mixing the DNA sample with orange dye, followed by loading onto the gel. For identifying aptamer DNA and probe DNA hybridization, electrophoresis was conducted on a 5% TBE gel under the conditions of 100 V for 45 min. Aptamer DNA binding affinity was tested using a 15% TBE-urea gel at 100 V for 1.5 h. Visualization of DNA bands in the gel was achieved using SYBR Safe DNA Gel Stain.

### Fluorescence resonance energy transfer (FRET) assay for validating the aptamer DNA-probe DNA complex

A FRET assay was employed to detect the release of probe DNA from an aptamer DNA-probe DNA hybridization complex. The aptamer DNA was labeled with BHQ-2 (Aptamer-Q) to quench the Cy5 fluorescence of the labeled probe DNA. A 1:1.25 molar ratio of probe DNA and aptamer-Q was prepared according to the same protocol described in Sect. 2.3. A 200 nM solution of the 20 µL probe DNA-Aptamer-Q complex was incubated with 100 µL of *E. coli* suspension at a concentration of 10^7^ CFU/mL for 1 h at 37 °C. After incubation, the *E. coli* cells were centrifuged at 2000 g for 10 min, and the supernatant was collected and diluted to a final concentration of 1 nM. Fluorescence spectra were measured with an excitation wavelength of 633 nm. All reactions were carried out in a reaction buffer (1 × PBST (0.05% v/v) containing 1% BSA, and 10 mM MgCl₂).

### Preparation of AuNPs-embedded magnetic beads (MB-AuNPs)

AuNPs were prepared using a slightly modified seeded growth method [29, 30]. A 150 mL solution of 2.6 mM sodium citrate was heated to 95 °C under vigorous stirring, followed by the addition of 1 mL of 20 mM HAuCl₄ solution. After 30 min, the solution was cooled to 90 °C and sequentially treated twice with 1 mL of 20 mM HAuCl₄, with a 30 min reaction time for each addition. The solution was then diluted by replacing 55 mL of the solution with 53 mL of DI water and 2 mL of 60 mM sodium citrate. This solution was used as the Au seed solution, and the growth process described above was repeated twice. As shown in Fig. S1, the shape and size distribution of the AuNPs were characterized using (a) UV–Vis spectrophotometry, (b) dynamic light scattering (DLS), and finally confirmed by (c) transmission electron microscopy (TEM). The size-confirmed AuNPs were washed three times with Tween 20 solution (0.1% v/v) and concentrated to 1 nM for further experiments.

MB-AuNPs were prepared using Au–S bond chemistry. In this work, commercially available superparamagnetic beads (ca. 1 µm in diameter) were employed in the preparation. Initially, 1 mL of 0.5 mg/mL carboxylic acid-activated magnetic beads were magnetically separated and resuspended in a solution containing 1 mM EDC and DTBA in pH 6.0 MES buffer to functionalize the carboxylic acid groups with thiol groups. The thiolated magnetic beads were then washed three times with Tween 20 solution (0.1% v/v) to remove unreacted chemicals. Subsequently, 200 μL of thiolated magnetic beads were magnetically separated and resuspended in 1 mL of 1 nM AuNPs, allowing the reaction to proceed for 24 h at room temperature under 1500 rpm. Finally, the AuNP-coated magnetic beads were washed six times with 1 × PBST (0.05% v/v) and Tween 20 solution (0.1% v/v) to remove non-specifically bound or unbound AuNPs and stored at a concentration of 0.5 mg/mL. The distribution and morphology of the AuNPs on the MB-AuNPs were characterized by scanning electron microscopy (SEM).

Ellman's tests for identification of thiol group functionalization were performed according to the manufacturer's instructions [31]. Briefly, the magnetic beads were washed five times with Ellman's reaction buffer (0.1 M sodium phosphate, pH 8.0, containing 1 mM EDTA) and resuspended in Ellman's reagent solution (80 μg/mL in Ellman’s Reaction Buffer). They were then incubated at room temperature for 15 min with thorough mixing. Following incubation, 100 µL of the reaction mixture was transferred into a 96-well microplate to measure absorbance at 412 nm.

### Preparation of capture DNA-conjugated MB-AuNPs (MB-AuNPs-cDNA)

First, 100 μM thiol-modified cDNA was treated with 10 mM TCEP at a molar ratio of 1:100 and allowed to react for 2 h at room temperature to reduce disulfide bonds. Then, 100 μL of 0.5 mg/mL MB-AuNPs were magnetically separated, and the supernatant was exchanged with 352 μL of Tween 20 solution (0.1% v/v) containing 1 M NaCl. After that, 8 μL of 50 μM TCEP-treated cDNA and 40 μL of 100 μM 6-MCH were sequentially added, and the mixture was allowed to react at 50 °C for 5 h under 1500 rpm. The resulting conjugates were washed three times with 1 × PBST (0.05% v/v) and stored at 4 °C until further use.

### SERS-based assay of *E. coli*

*E. coli* cell suspension was serially diluted to prepare a range of concentrations. Then, 10 μL of 200 nM aptamer DNA-probe DNA was added to 20 μL of each *E. coli* dilution. Subsequently, 30 μL of 0.125 mg/mL MB-AuNPs-cDNA was magnetically separated, and the previous mixture was added to the separated MB-AuNPs-cDNA. The combined mixture was incubated at 37 °C for 6 h. Following incubation, the complexes were washed three times with 1 × PBST (0.05% v/v) and resuspended in 1 × PBS. The resulting complexes from the microtube were transferred to a capillary tube for Raman measurement. Raman spectra were collected with a 10 s exposure time, with measurements repeated three times and averaged (n = 3). All reactions were carried out in a reaction buffer (1 × PBST (0.05% v/v) containing 1% BSA and 10 mM MgCl₂). Selectivity tests for other bacterial species (*S. aureus* and *MRSA*) were performed by the same method described above.

### Instrumentation

Dynamic Light Scattering (DLS) measurements were performed using a Nano-ZS90 instrument (Malvern, UK). TEM images were acquired with a JEM-F200 (JEOL, USA). SEM was conducted using a SIGMA 300 (Zeiss, Germany). The size distribution of AuNPs was calculated using ImageJ software. UV–Vis spectra were acquired with a UV-2600 (Shimadzu, Japan). Fluorescence spectra were acquired with an RF-6000 (Shimadzu, Japan). Gel electrophoresis was conducted using a Mini-PROTEAN Tetra Cell (Bio-Rad, USA) and imaged with a Gel Documentation System (Corning, USA). All Raman spectral data were obtained using a Renishaw inVia Raman microscope (Renishaw, New Mills, UK). A He–Ne laser at 632.8 nm was used as the excitation source, and the power of the laser after passing through the lens was 9.8 mW. The Raman scattering signals were measured by focusing the laser spot on the tube using a 20 × objective lens (N.A. 0.40) and collected with a charge-coupled device (CCD) camera at a spectral resolution of 1 cm⁻^1^. Baseline correction and spectral smoothing for each Raman spectrum were performed using Renishaw WIRE 4.0 software. All other data processing was conducted using Origin 2017 and Excel.

### Assessment of efficacy for clinical samples

To assess the clinical applicability of the SERS-based aptasensor, a total of 21 clinical urine samples—comprising 11 UTI-positive specimens and 10 negative controls—were analyzed. To minimize matrix effects, the clinical samples were washed three times with 1 × PBST (2000 rcf, 10 min) prior to testing. These PBST washing steps were implemented to eliminate potential interfering substances—including proteins, nucleic acids, and chemicals—that might be present in patient urine samples. After the washing steps, the bacteria were resuspended in the same volume of buffer, so any change in bacterial concentration due to volume changes is considered to be minimal. Although some bacterial loss may occur during washing, this does not exert a critical impact on the accuracy of positive or negative classification. Additionally, since matrix effects can vary significantly among individuals, applying a normalization or correction factor based on a single standard was deemed inappropriate. Therefore, aside from baseline correction and smoothing for the Raman spectra, no additional normalization or correction was performed. The assay protocol was identical to that described in Sect. 2.7, except that the reaction buffer consisted of 1 × PBST (0.05% v/v) supplemented with 0.1% casein and 10 mM MgCl₂. The study protocol for the use of clinical samples was approved by the Institutional Review Board (IRB) of Chung-Ang University Hospital.

## Results and discussion

### Strategy for SERS-based assays of *E. coli* using MB-AuNPs substrates

**Fig. 1:**
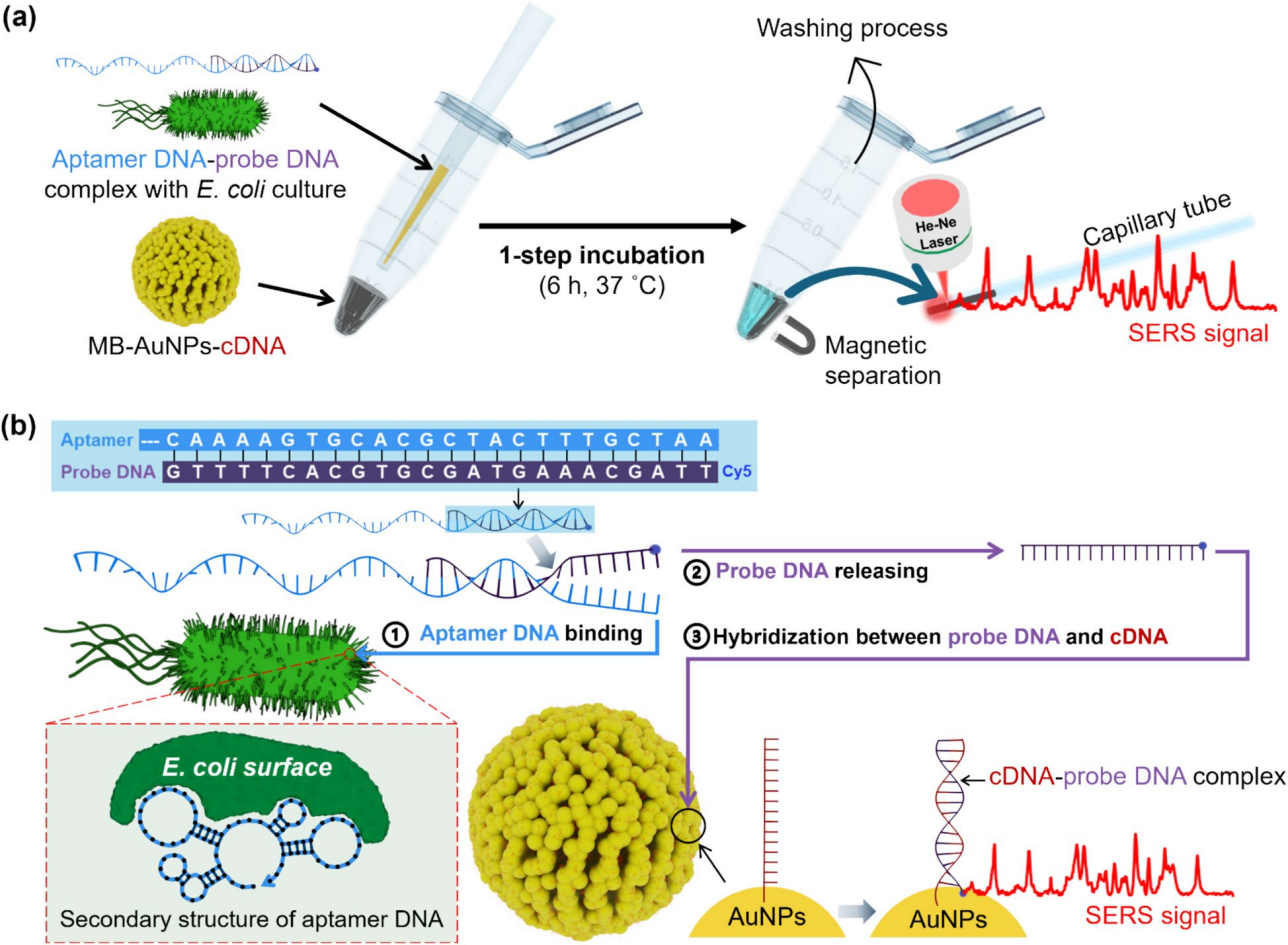
**a** Schematic illustration of the SERS-based aptasensor. AuNP-embedded MBs are used for detecting *E. coli*. (**b**) Working principle of the SERS-based aptasensor. Aptamer DNA–probe DNA hybridization complexes interact with *E. coli*, causing the probe DNA strands to release as the aptamer DNA binds to bacterial surface proteins. Liberated probe DNA hybridizes with capture DNA immobilized on MB-AuNPs-cDNA conjugates through complementary base pairing. A strong SERS signal is generated when Cy5 molecules on the probe DNA are brought into close proximity to the AuNP surface

Figure 1a shows a schematic illustration of the SERS-based aptasensor using AuNP-embedded MBs (MB-AuNPs) for the detection of *E. coli*. While conventional bacteria assay methods such as culture and colony counting or RT-PCR involve multi-step processes, the SERS-based aptasensor assay developed in this study is a one-step process. Initially, a partial dsDNA formed by hybridization of aptamer DNA (88 mer in Table 1) and Cy5-labeled probe DNA (24 mer in Table 1), target *E. coli*, and MB-AuNPs labeled with capture DNA (cDNA, 24 mer in Table 1) were placed in a microtube and allowed to react at 37 °C for 6 h. After the reaction, the supernatant was removed using a magnetic bar, washed with a buffer solution, and then transferred to a capillary tube for Raman measurements. Figure 1b illustrates the principle of the SERS-based aptasensor. When the aptamer DNA-probe DNA hybridization complexes encountered *E. coli*, the probe DNA strands were released from the complex as the aptamer DNA bound to the surface protein of *E. coli*. The released probe DNAs then hybridized with the cDNA immobilized on the MB-AuNPs-cDNA through DNA hybridization. Because the Cy5 molecules attached to the probe DNAs were in close proximity to the AuNP surface, a strong SERS signal could be detected.

### Fabrication of MB-AuNPs substrates and their characterization

**Fig. 2:**
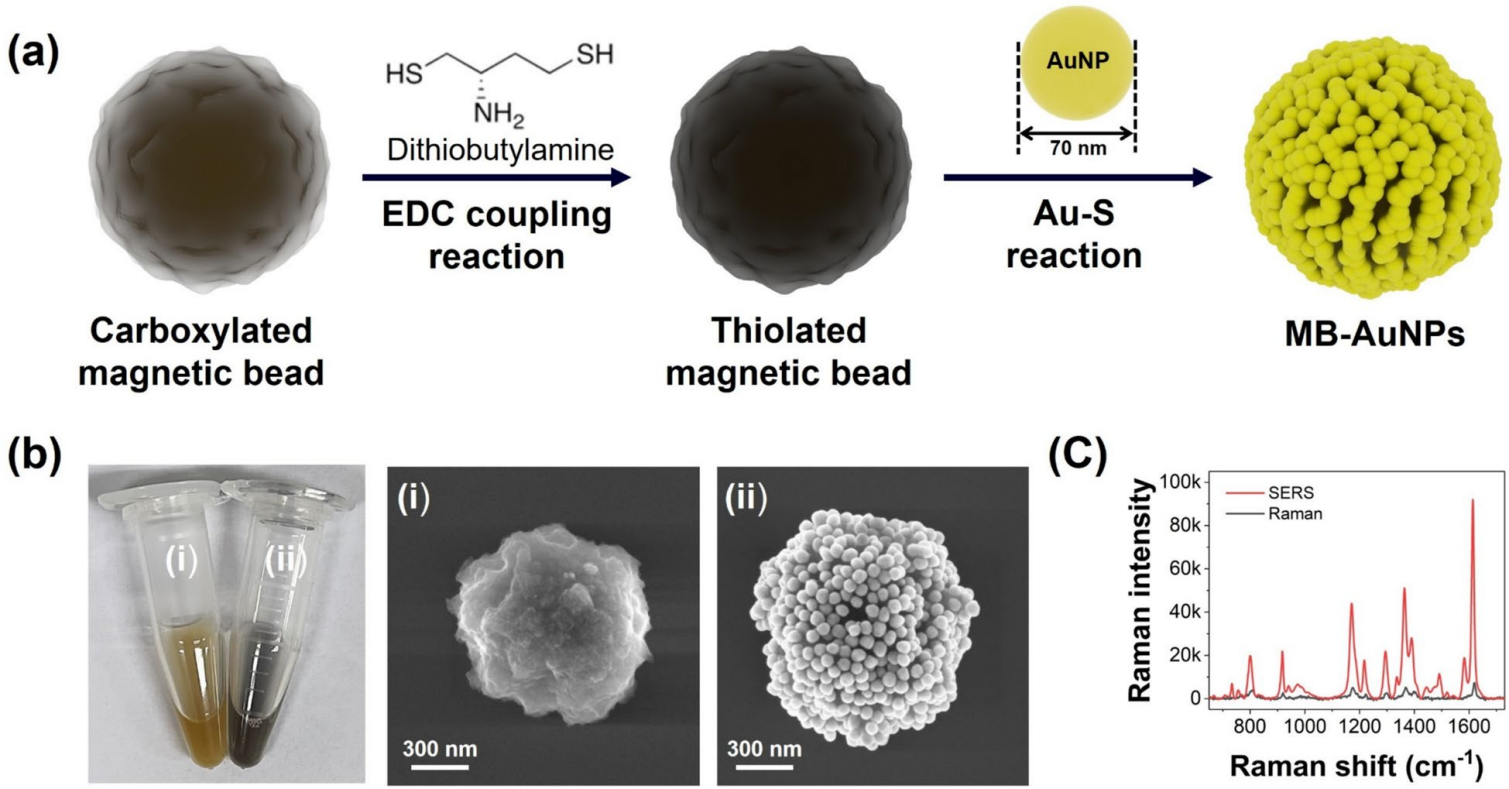
**a** Preparation of MB-AuNPs through surface modification of MBs with thiol groups and subsequent attachment of AuNPs via Au–S bonds. Carboxylated MBs were functionalized with thiol groups using dithiobutylamine via an EDC coupling reaction. **b** SEM images of (i) pristine MBs and (ii) MBs conjugated with 70 nm-sized AuNPs. The color of the MB solution changed from brown to deep grey after AuNP conjugation. **c** Raman spectrum of 10 μM MGITC (black) and SERS spectrum of 100 nM MGITC on MB-AuNPs (red). Raman spectra were collected with a 10 s exposure time, with measurements repeated three times and averaged (n = 3)

To induce strong SERS signals from cDNA-probe DNA hybridization complexes and effectively separate them, it is necessary to synthesize MB-AuNPs that can generate strong hot spots on the surface. To prepare MB-AuNPs, magnetic beads were modified with thiol groups and AuNPs were attached through Au–S bond. Carboxylated MBs were functionalized with thiol groups using dithiobutylamine (DTBA) via an EDC coupling reaction as shown in Fig. 2a. Ellman’s test was performed to confirm the functionalization of thiol groups on the magnetic beads (Fig. S2). Ellman's test is a colorimetric assay used to detect and quantify free sulfhydryl (–SH) groups, such as those found in thiol-containing compounds. The assay involves the reaction of sulfhydryl groups with DTNB, producing a yellow-colored product, TNB, which has an absorbance peak at 412 nm (Fig. S2a). Only beads treated with both EDC and DTBA displayed a discernible yellow color change, as shown in the inset of Fig. S2b. Additionally, only the EDC/DTBA-treated bead exhibited the significant absorbance at 412 nm comparable to original DTBA solution. Subsequently, conjugation of 70 nm-sized AuNPs to the thiolated beads were conducted via Au–S bond. The successful conjugation of 70 nm-sized AuNPs onto magnetic beads was verified by SEM images in Fig. 2b and Fig. S3. Densely packed AuNPs and numerous nanogaps between AuNPs were constructed. The color of the magnetic bead solution was changed from brown to deep grey after the AuNPs conjugation as shown in Fig. 2b. The SERS performance of MB-AuNPs was evaluated using MGITC as a Raman reporter. The normal Raman spectrum of 10 μM MGITC was compared with the SERS spectrum of 100 nM MGITC on MB-AuNPs. As seen in Fig. 2c, the SERS peak intensity from MB-AuNPs was significantly stronger, even though the concentration of MGITC was 100 times lower, confirming the SERS capabilities of MB-AuNPs. The enhancement factor (EF) was estimated to be 1.2 × 10^3^ by the following equation [32].

$$EF=\frac{{I}_{MGITC-SERS}}{{I}_{MGITC-Raman}}.\frac{{C}_{MGITC-Raman}}{{C}_{MGITC-SERS}}$$

where *I*_*MGITC-SERS*_ and *I*_*MGITC-Raman*_ represent the SERS and normal Raman peak intensities of MGITC corresponding to the stretching vibration of the C–C and N-phenyl ring mode (1615 cm^−1^), obtained from the MGITC-reacted MB-AuNPs solution and the MGITC solution alone, respectively. Both experiments were performed using glass capillary tubes under identical measurement conditions. *C*_*MGITC-SERS*_ and *C*_*MGITC-Raman*_ denote the concentrations of MGITC in the MB-AuNPs and MGITC solutions, respectively.

### Preparation of MB-AuNPs substrate functionalized with cDNAs

**Fig. 3:**
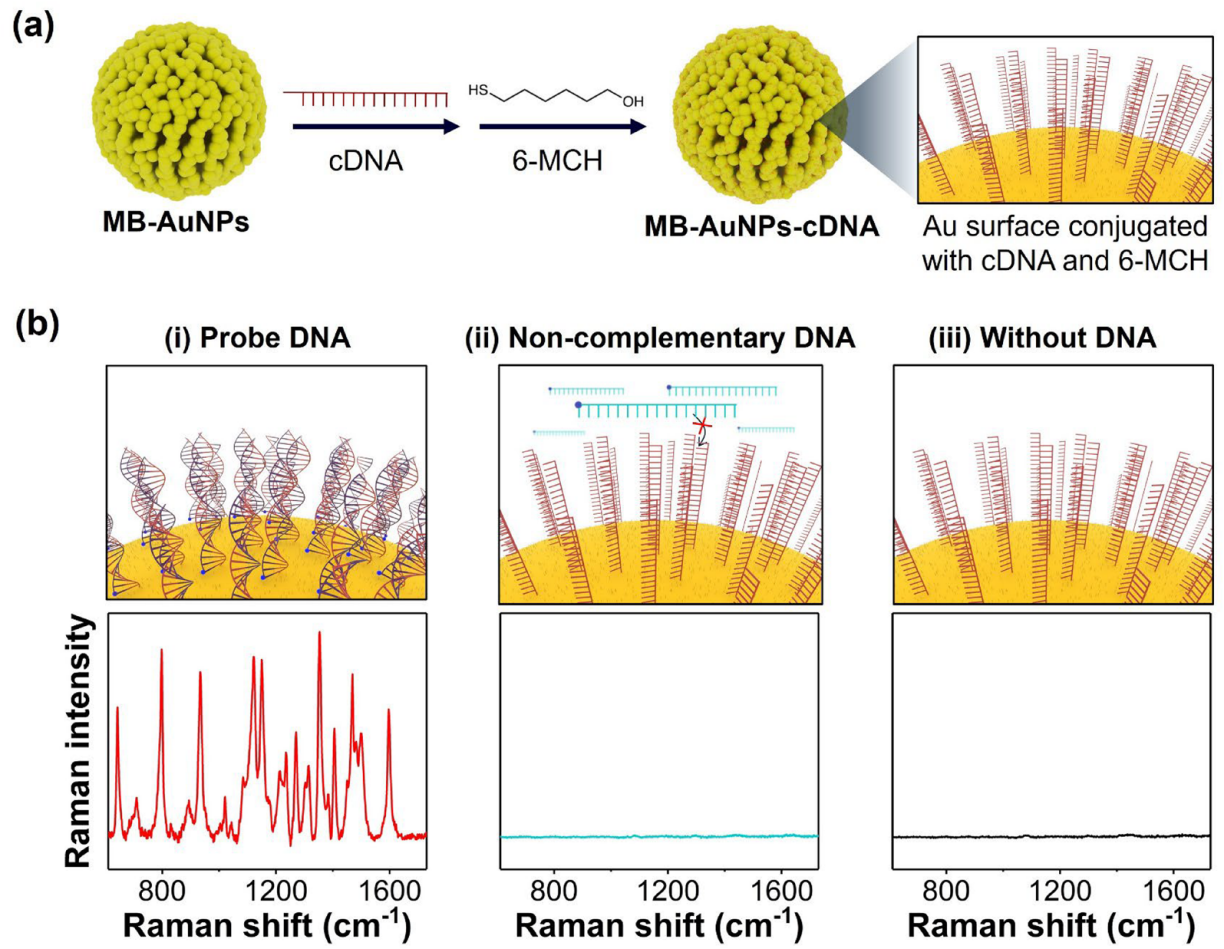
**a** Conjugation of cDNAs onto the surface of MB-AuNPs. cDNA strands were immobilized onto the MB-AuNPs via Au–S bonds, while unreacted sites were blocked with 6-MCH. **b** DNA hybridization tests of MB-AuNPs-cDNA with (i) complementary DNA, (ii) non-complementary DNA, and (iii) no DNA. All Raman spectra were measured three times and averaged (n = 3)

MB-AuNPs functionalized with capture DNA (MB-AuNPs-cDNA) were prepared following the process shown in Fig. 3a. cDNAs were immobilized onto the MB-AuNPs via Au–S bonds, and unreacted sites were simultaneously blocked with 6-MCH. Figure 3b demonstrates that the prepared MB-AuNPs-cDNA specifically captured the complementary probe DNAs. Both Cy5-labeled complementary probe DNAs and non-complementary DNAs were reacted with MB-AuNPs-cDNA, and the Raman signals were measured. Only the MB-AuNPs-cDNA that reacted with the probe DNAs exhibited the characteristic SERS spectrum of Cy5 molecules. This result highlights the ability of MB-AuNPs-cDNA to selectively capture the complementary probe DNA sequences, clearly distinguishing them from non-complementary DNAs.

To quantify the number of cDNA molecules conjugated to MB-AuNPs and the number of probe DNA molecules hybridized, we performed fluorescence-based experiments. First, we quantified the amount of cDNA bound to MB-AuNPs using a dithiothreitol (DTT)-based desorption method [33]. Cy5-labeled cDNAs at various concentrations were prepared and a standard calibration curve was established as *y* = 4.3*x* + 12.99 from their fluorescence measurement data. After conjugating MB-AuNPs with cDNA-Cy5, we used a high concentration of DTT (1 M) to detach the surface-bound cDNAs. The average fluorescence intensity of the recovered solution was measured to be 8324.0, corresponding to 1.92 nM according to the calibration curve. Since the sample was diluted 100-fold for fluorescence measurement, the original concentration was calculated to be 192 nM. From this result, we estimate that approximately 19.2% of the added 1 μM cDNA was conjugated to the MB-AuNPs. Based on supplier information (approximately 9.5 × 10⁹ beads/mL at 10 mg/mL), we estimated that each MB-AuNPs was conjugated with an average of approximately 9.6 × 10^14^ cDNA molecules.

Additionally, we quantified the amount of probe DNA hybridized to cDNA. Probe DNA was prepared at various concentrations and their fluorescence signals were measured to generate a standard calibration curve (*y* = 18.54*x* + 3.24). Probe DNA at the same concentration used in the *E. coli* assay (66.7 nM) was then reacted with the MB-AuNPs-cDNA complex (6 h, 37 °C). The supernatant was collected, diluted 3.33-fold, and its fluorescence intensity was measured, yielding a value of 78.7. Using the calibration curve and dilution factor, the remaining probe DNA concentration was calculated to be 13.7 nM. This indicates that approximately 53.0 nM of the added 66.7 nM probe DNA (79.5% of the total) participated in the hybridization reaction. All fluorescence experiments were repeated three times (n = 3), and the error bars shown in the graphs represent the corresponding standard deviations. The reported fluorescence intensities represent average values. Fluorescence measurements were performed with an excitation wavelength of 633 nm and an emission wavelength of 665 nm.

### Confirmation of binding between aptamer DNA-probe DNA complex and bacteria

DNA gel electrophoresis was performed to verify the selectivity of the aptamer DNA. Aptamer DNAs were incubated with different bacterial species (*E. coli* and *S. aureus*), and bacteria were removed by centrifugation. The remaining aptamer DNAs in the supernatant were then analyzed by electrophoresis. As shown in Fig. S4a, no bands appeared for the aptamer DNAs that had reacted with *E. coli*, indicating specific binding to *E. coli*. On the other hand, in the case of *S. aureus*, the electrophoretic band was still observed even after reacting with aptamer DNAs, indicating that no reaction occurred between the bacteria and aptamer DNAs. To confirm the formation of the aptamer DNA-probe DNA hybridization complexes, additional electrophoresis was conducted for the probe DNA, aptamer DNA, and aptamer DNA-probe DNA complexes. Fig. S4b shows that the aptamer DNA-probe DNA complexes migrated more slowly, forming a higher band due to their larger molecular weight compared to the individual aptamer DNA and probe DNA bands.

Fluorescence Resonance Energy Transfer (FRET) assays were also conducted to confirm the release of probe DNAs from the aptamer DNA-probe DNA hybridization complexes upon interaction with *E. coli* (Fig. S5). An aptamer DNA (Aptamer-Q in Table 1) labeled with a quencher molecule (BHQ-2) was used to enable FRET quenching of the fluorescence emission for Cy5, which was labeled at the terminal of probe DNA. Because the FRET mechanism is highly dependent on the distance between the donor (Cy5) and the acceptor (BHQ-2) molecules, the close proximity between Cy5 and BHQ-2 in the aptamer DNA-probe DNA complex results in FRET quenching, significantly reducing the fluorescence emission of Cy5. Fig. S5a presents a schematic illustration of the process, showing how Cy5 fluorescence is restored when the aptamer DNA binds to *E. coli*. This binding event causes the aptamer DNA-probe DNA complex to dissociate, increasing the distance between Cy5 and BHQ-2 and thereby weakening the quenching effect. Consequently, the fluorescence emission of Cy5 is recovered. As depicted in Figs. S5b and S5c, significant fluorescence recovery was observed when the aptamer DNA encountered *E. coli*, confirming that the aptamer DNA’s binding to *E. coli* triggered the release of the probe DNA. This result highlights the aptamer DNA’s specificity and supports the proposed mechanism in which probe DNA is released upon aptamer DNA binding to *E. coli*.

### Quantitative analysis and selectivity test of *E. coli* using a SERS-based aptasensor

**Fig. 4:**
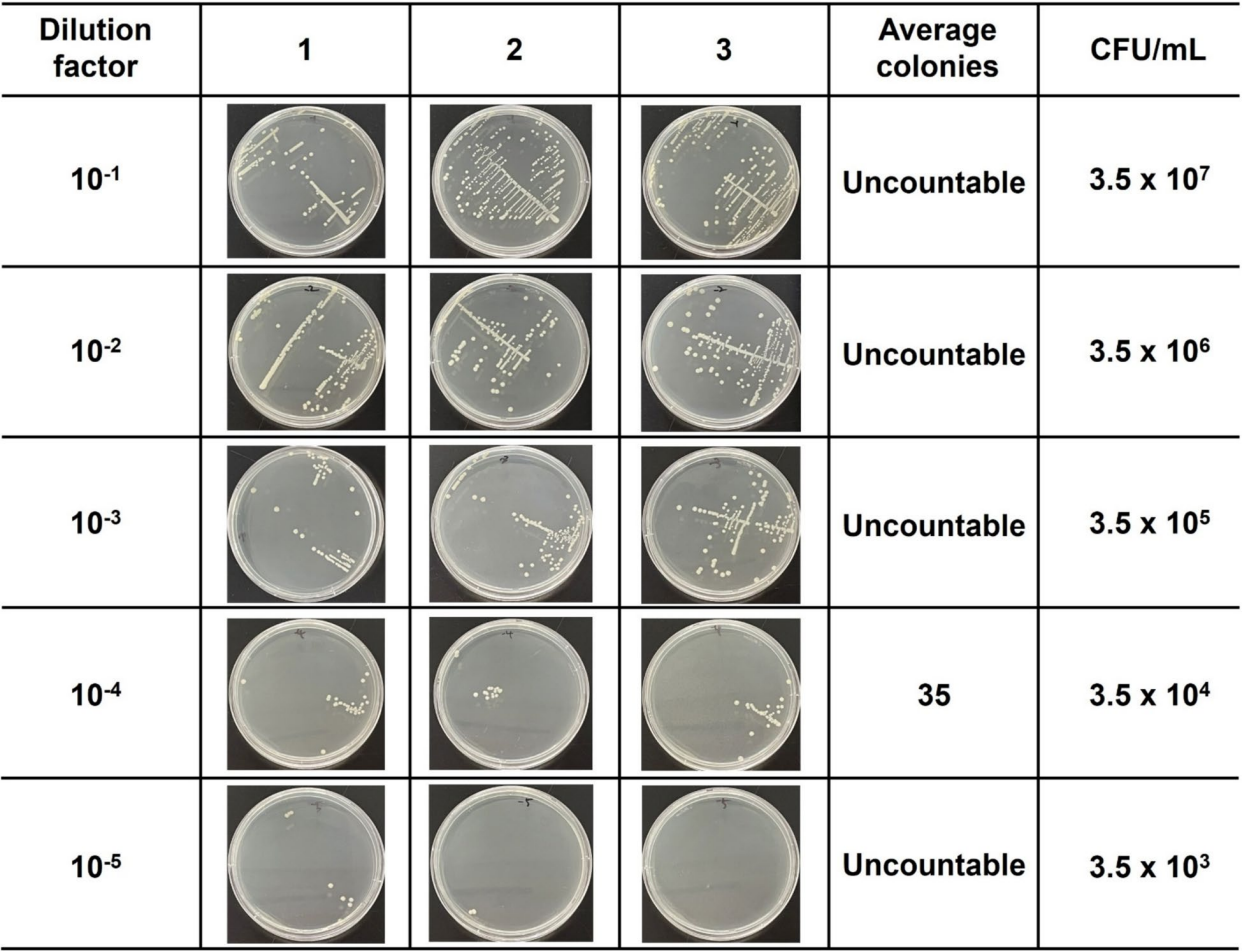
Colony counting results for *E. coli* culture dilutions. Using a 1 µL loop, colony counts exceeding 100, corresponding to 10^5^ CFU/mL, indicate concentrations above the clinical cutoff for UTIs. At dilution levels of ≤ 10⁻^3^, more than 100 colonies were observed, confirming concentrations greater than 10^5^ CFU/mL. In contrast, at a dilution of 10⁻^4^, the average colony count was 35, which falls below the clinical cutoff value

Figure 4 shows the colony counting results for various dilutions of the *E. coli* culture. In colony counting performed with a 1 µL loop, observing more than 100 colonies corresponds to 10^5^ CFU/mL, which is the clinical cutoff value for UTIs. For example, when the dilution factor is 10⁻^3^ or lower, over 100 colonies are formed, confirming a concentration exceeding 10^5^ CFU/mL. At a dilution factor of 10⁻^4^, however, the average number of colonies is 35, which is below the cutoff value. Since the dilution factor of 10⁻^4^ yields a colony count between 30 and 300, which is considered the optimal range for accurate counting. The concentration of the original *E. coli* culture was determined by this colony counting method and subsequently compared with SERS-based bacteria assay data.

**Fig. 5:**
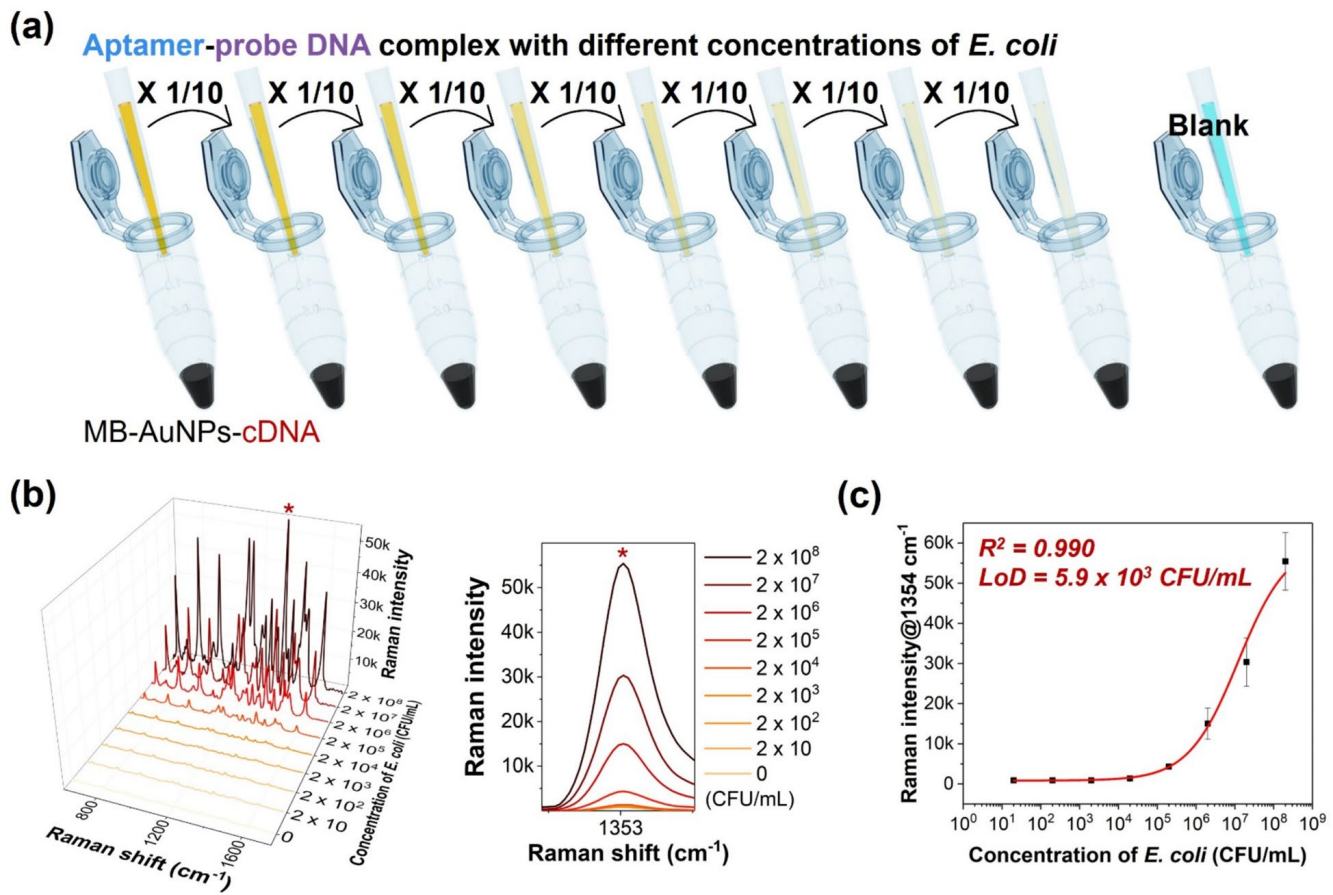
Illustration of SERS-based assay data for the quantitative analysis of *E. coli*. **a** *E. coli* cell suspensions were serially diluted tenfold increments to facilitate quantitative analysis. **b** SERS spectra obtained for target DNA hybridized to MB-AuNPs-cDNA at increasing *E. coli* concentrations ranging from 0 to 2 × 10^8^ CFU/mL, alongside Raman peak intensity variations at 1354 cm^−1^. All Raman spectra were measured three times and averaged (n = 3). **c** A corresponding calibration curve generated based on the Raman peak intensity variations

Figure 5a illustrates the SERS-based assay data for the quantitative analysis of *E. coli. E. coli* cell suspensions, prepared using the same method as in the colony counting experiments, were serially diluted tenfold to conduct the quantitative analysis. As shown in Fig. 5b, stronger Raman signals from Cy5, which is attached to the probe DNA hybridized to the MB-AuNPs-cDNA, were observed with increasing *E. coli* concentrations. The characteristic Raman peak intensity at 1354 cm⁻^1^ was used to construct a calibration curve using a four-parameter logistic (4PL) equation. As shown in Fig. 5c, a good correlation coefficient (R^2^ = 0.990) was obtained, and the limit of detection (LoD) was calculated to be 5.9 × 10^3^ CFU/mL, which is well below the UTI cutoff value.

**Fig. 6:**
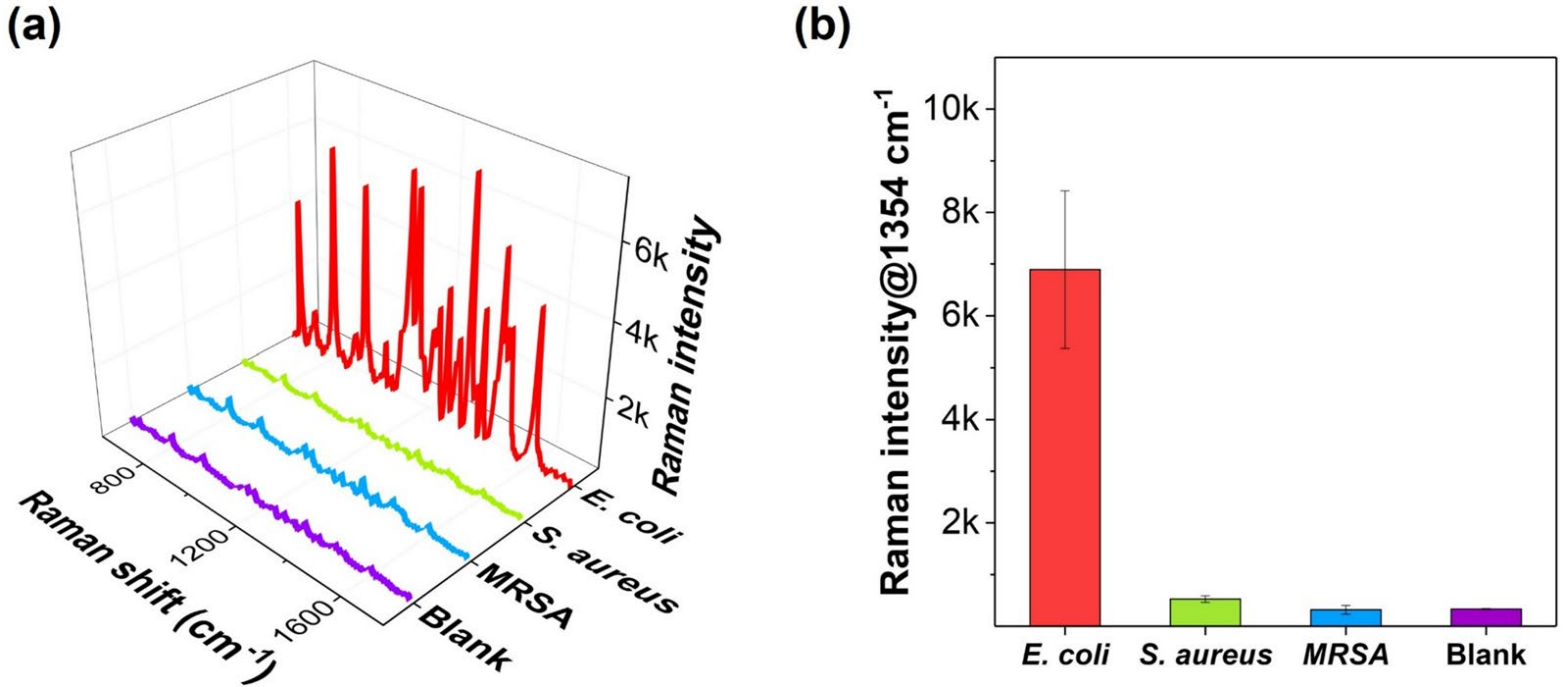
Selectivity test of the SERS-based aptasensor for *E. coli*, *S. aureus*, and methicillin-resistant *S. aureus* (MRSA), each at a concentration of 2 × 10⁷ CFU/mL. **a** SERS spectra corresponding to the three bacterial types. All Raman spectra were measured three times and averaged (n = 3). **b** Histogram showing Raman peak intensities at 1354 cm⁻^1^ for each bacterium

To test the selectivity of the SERS-based aptasensor developed in this study for *E. coli*, a SERS-based assay was performed on three types of bacteria—*E. coli*, *S. aureus*, and *methicillin-resistant Staphylococcus aureus* (*MRSA*)—all at the same concentration of 2 × 10⁷ CFU/mL. Figure 6a presents the SERS spectra for the three types of bacteria, while Fig. 6b compares the histogram of Raman peak intensities at 1354 cm⁻^1^ for each bacterium. As shown in this figure, only *E. coli* produced a strong Raman signal, whereas the other bacteria at the same concentration did not exhibit significant responses. This confirms that the developed SERS-based aptasensor demonstrates high specificity for *E. coli*.

### Reproducibility test of SERS-based assays for *E. coli*

To assess repeatability, we targeted *E. coli* using five independent sample preparations, each at a concentration of 2 × 10^5^ CFU/mL and acquired the Raman signal three times per sample. The %RSD was determined to be 9.6%. Fig. S6a shows the repeatability of the Raman signal intensity at 1354 cm^−1^ obtained from the five sample preparations. In addition, to evaluate day-to-day reproducibility, we performed SERS measurements of *E. coli* at the same concentration on three consecutive days under identical experimental conditions. In this case, the %RSD was determined to be 6.5%. Fig. S6b shows the day-to-day reproducibility of Raman signal intensity at 1354 cm^−1^. As the %RSD values for both experiments described above were below 10%, these results confirm that the SERS-based aptasensor developed in this study exhibits statistically high precision in terms of repeatability and day-to-day reproducibility. All SERS measurements were conducted under the conditions described in the Experimental Section (9.8 mW laser power, 20 × objective lens, and 10 s exposure time).

To further validate the signal reproducibility, we performed single-particle SERS experiments to evaluate the distribution and uniformity of SERS signal intensity at the level of individual MB-AuNPs. First, silicon wafer was washed with acetone and ethanol, dried with N₂ gas. Then 10 μL of MB-AuNPs at a concentration of 4.8 × 10⁷ beads/mL was drop-cast onto the silicon wafer and dried in a 70 °C oven for 30 min. Subsequently, 10 μL of 1 μM MGITC ethanol solution was drop-cast and further dried for 30 min in the same oven to prepare the sample for Raman analysis.

A Raman mapping image at the single-particle level was measured using a Raman microscope (Fig. S7a). Through Raman mapping, individual MB-AuNPs exhibiting the SERS peak intensity of MGITC (1615 cm⁻^1^) were identified. The mapping was performed over a 3 × 3 μm^2^ area at intervals of 0.5 μm. The laser spot size was calculated to be approximately 1.404 μm according to the diffraction limit formula (1.22λ/N.A.). A 50 × objective lens with a numerical aperture of 0.55 and a 632.8 nm He–Ne laser were used. The laser power after passing through the lens was measured at 115.7 μW, and the exposure time was set to 0.1 s. Raman mapping was also performed under the same conditions for a total of 100 individual particles. The Raman intensity shown in Fig. S7b represents the average of the three strongest SERS peaks (1615 cm⁻^1^) within the mapping area of each individual particle, and the error bars indicate the standard deviation for these values. Based on this analysis, the mean Raman signal for the 100 particles was calculated to be 3762.4 ± 919.0, with a %RSD of 24.4%.

The relatively high %RSD is thought to result from the variation in the number of MGITC molecules adsorbed onto individual MB-AuNPs and the inherent limitations in achieving uniform formation of plasmonic hot spots among the gold nanoparticles distributed on the surface of the magnetic beads. However, in practical analyses using the SERS-based aptasensor, signals are averaged (ensemble-averaged) from multiple MB-AuNPs present within the laser focal volume, rather than from a single particle. This ensemble averaging mitigates such single-particle-level variability, which is consistent with the previously reported precision and reproducibility results, in which high precision was achieved (%RSD < 10%).

### Efficacy evaluation of clinical samples

**Fig. 7:**
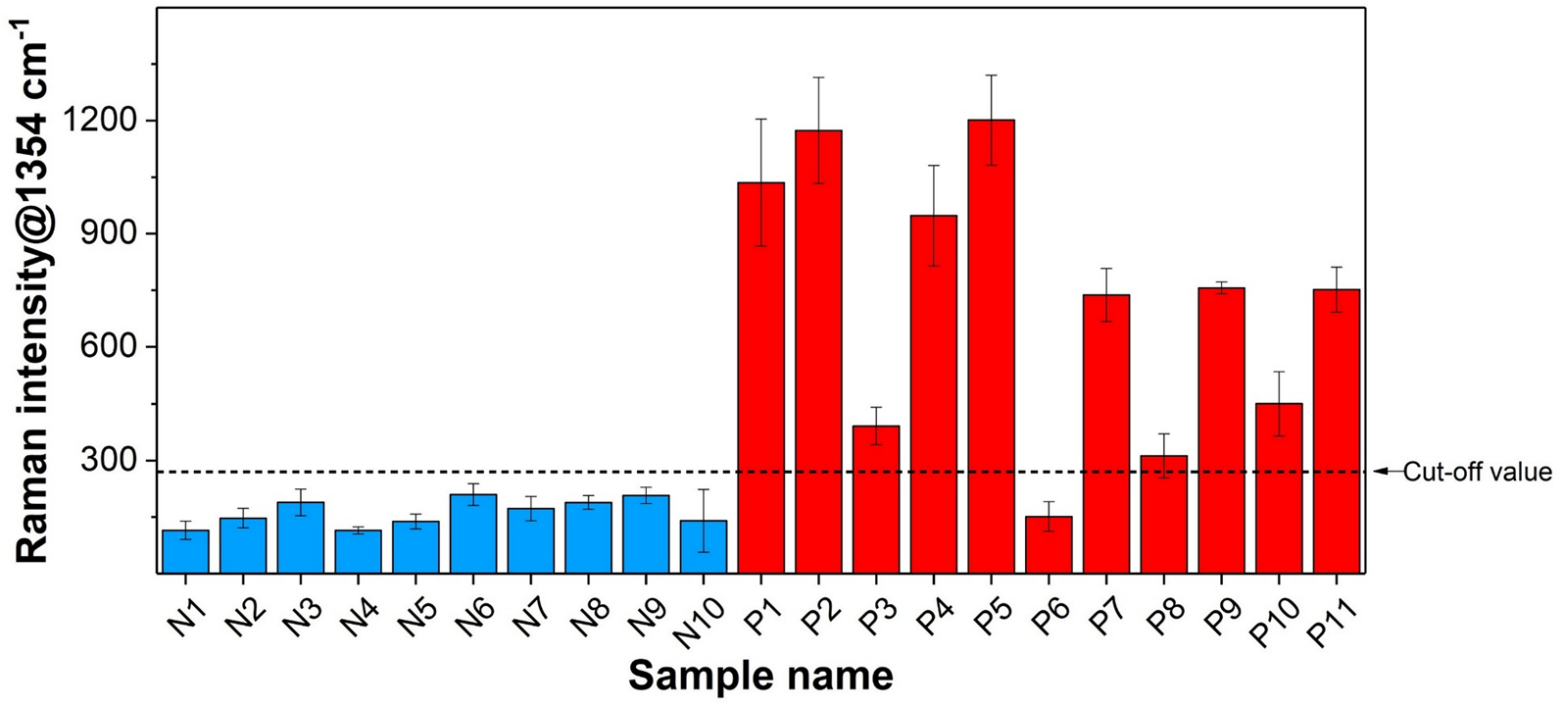
Clinical test results of the SERS-based aptasensor using 21 patient urine samples. P1–P11 represent UTI-positive samples confirmed to contain *E. coli* via standard urine culture, while N1–N10 are negative controls. The cut-off value was determined based on the mean + 3σ of the Raman intensities at 1354 cm^−1^ from the negative controls

The clinical performance of the SERS-based aptasensor was evaluated using 21 patient-derived urine samples, comprising 11 UTI-positive samples (confirmed via urine culture to contain *E. coli*) and 10 negative controls. A significant difference in Raman peak intensities at 1354 cm⁻^1^ was observed between positive and negative samples (Fig. 7, Table S1), with the assay’s diagnostic criteria established using a Raman cut-off value derived from the 1354 cm⁻^1^ intensities of the negative controls (Table S2). The SERS assay exhibited 100% concordance with urine culture results except for the P6 sample, and notably correctly identified the P3 sample as positive despite its bacterial count falling below the clinical threshold (< 10^5^ CFU/mL). Compared to urine culture, the aptasensor demonstrated excellent diagnostic performance, achieving 100% sensitivity, 91% specificity, 95% accuracy, and 100% precision (Table S2), highlighting its potential for clinical applications requiring high sensitivity.

## Conclusion

In this study, we developed a SERS-based aptasensor for the rapid and sensitive detection of *E. coli*, the most frequent causative pathogen for UTIs. MB-AuNPs-cDNA was employed as a SERS substrate, which possesses both superparamagnetic and SERS-active properties, enabling the simple detection of *E. coli* in a single-step manner without the need for additional SERS nanotags. The assay mechanism involves the use of an aptamer DNA-probe DNA complex, where the aptamer DNA specifically binds to *E. coli*, causing the release of the probe DNA. The released probe DNA is then captured by MB-AuNPs-cDNA through a DNA hybridization reaction, and the SERS signal from the Cy5 label at the probe DNA terminus is used for *E. coli* detection. Quantitative analysis using *E. coli* cell suspensions cultured under standard urine culture protocols yielded a reliable calibration curve (R^2^ = 0.990) with a detection limit of 5.9 × 10^3^ CFU/mL, which is well below the clinical cutoff value. Selectivity tests using other bacterial species were also performed, confirming that the *E. coli* aptasensor worked specifically without any cross-reactivity. The clinical evaluation demonstrated excellent agreement between the SERS-based aptasensor and conventional urine culture results across all 21 patient samples, achieving a sensitivity of 100%, specificity of 91%, accuracy of 95%, and precision of 100%. Notably, these results were obtained with a total assay time of  6 h, without the need for bacterial culture or nucleic acid amplification.

We have summarized and compared the sensor performances reported in previous studies for *E. coli* detection related to UTI diagnosis in Table S3. As shown in this table, although methods such as immunoluminescence, LFA strip, or RT-PCR can shorten the assay time, the assay still requires two-step processes, and it can be observed that the colorimetric assay method has limitations in sensitivity. Collectively, these findings support the potential of this method as a promising alternative to standard urine culture for UTI diagnosis. However, since urine samples exhibit considerable variability in matrix effects due to individual health status, diet, medication, and other factors, the current analysis, which utilized 21 clinical samples (encompassing both positive and negative cases) may be statistically underpowered. To achieve more robust clinical validation, additional studies involving a larger cohort of clinical samples—specifically at least 50–100 positive and negative samples each—are necessary, and this study is currently in progress.

## Supplementary Information

Supplementary Material 1.

## Data Availability

The datasets used and/or analyzed during the current study are available from the corresponding author on reasonable request.
