# Supplementary material for: SERS-based aptasensor for culture-free detection of Escherichia coli in urinary tract infection diagnosis
Source: Nano Converg. 2025 Aug 21;12:40. doi: 10.1186/s40580-025-00506-0 (PMC12370591; doi:10.1186/s40580-025-00506-0)
Supplement: Supplementary file 1 — Supplementary Material 1. [file 40580_2025_506_MOESM1_ESM.docx]

***Supplementary Information***

**SERS-based aptasensor for culture-free detection of *Escherichia coli* in urinary tract infection diagnosis**

Kihyun Kim^1^, Sohyun Park^1^, Suyoung Kang^1^, Mi-Kyung Lee^2^, Lingxin Chen^3,^*, Jaebum Choo^1,^*

^1^ *Department of Chemistry, Chung-Ang University, Seoul 06974, South Korea*

^2^ *Department of Laboratory Medicine, Chung-Ang University College of Medicine, Seoul 06973, South Korea*

^3^ *CAS Key Laboratory of Coastal Environmental Processes and Ecological Remediation, Yantai Institute of Coastal Zone Research, Yantai, 264003, China*

**
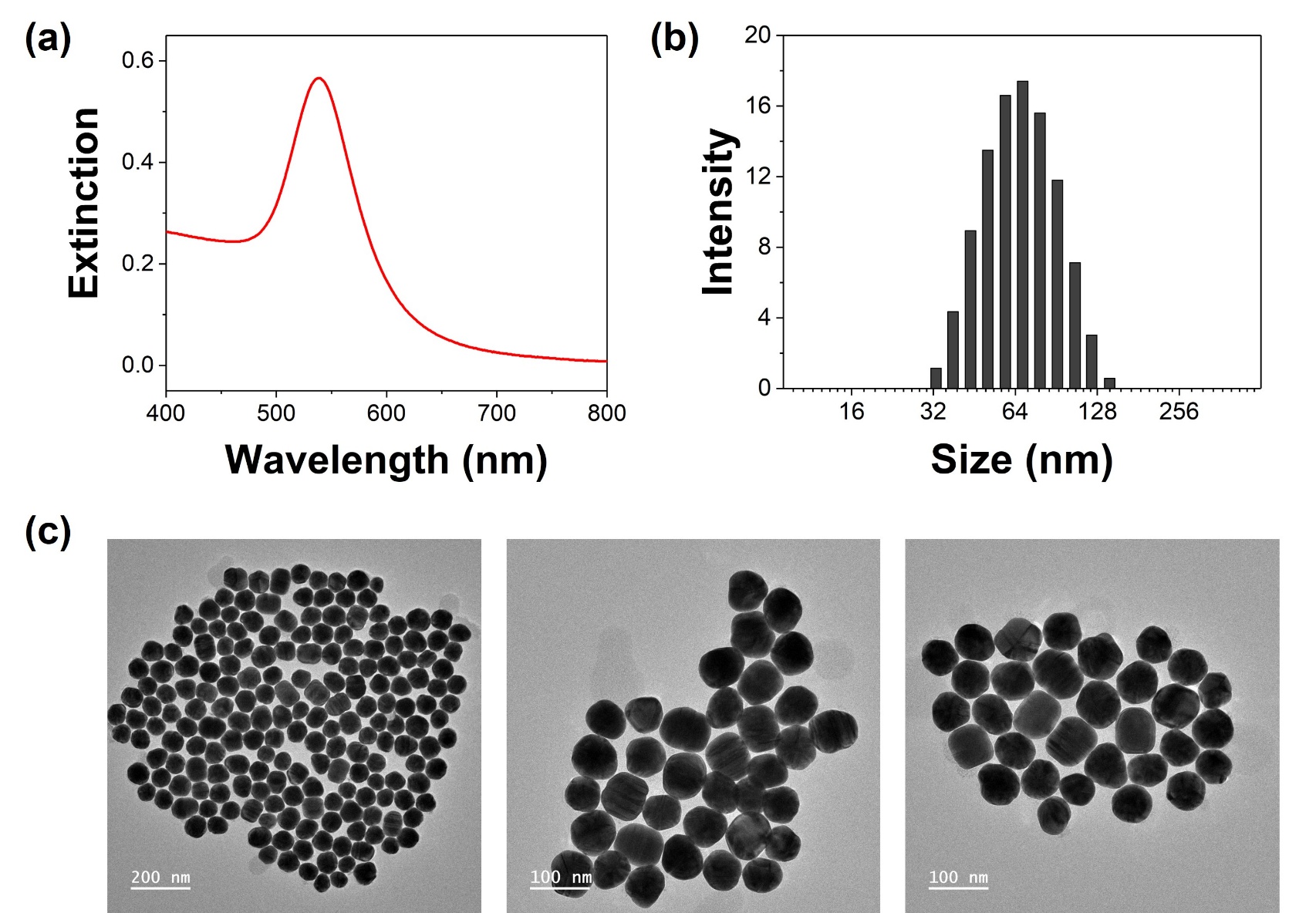
**

**Fig. S1.** Characterization of 70-nm sized gold nanoparticles synthesized using the seeded growth method. (a) UV-vis extinction spectrum, (b) DLS distribution, and (**c)** TEM images.

**
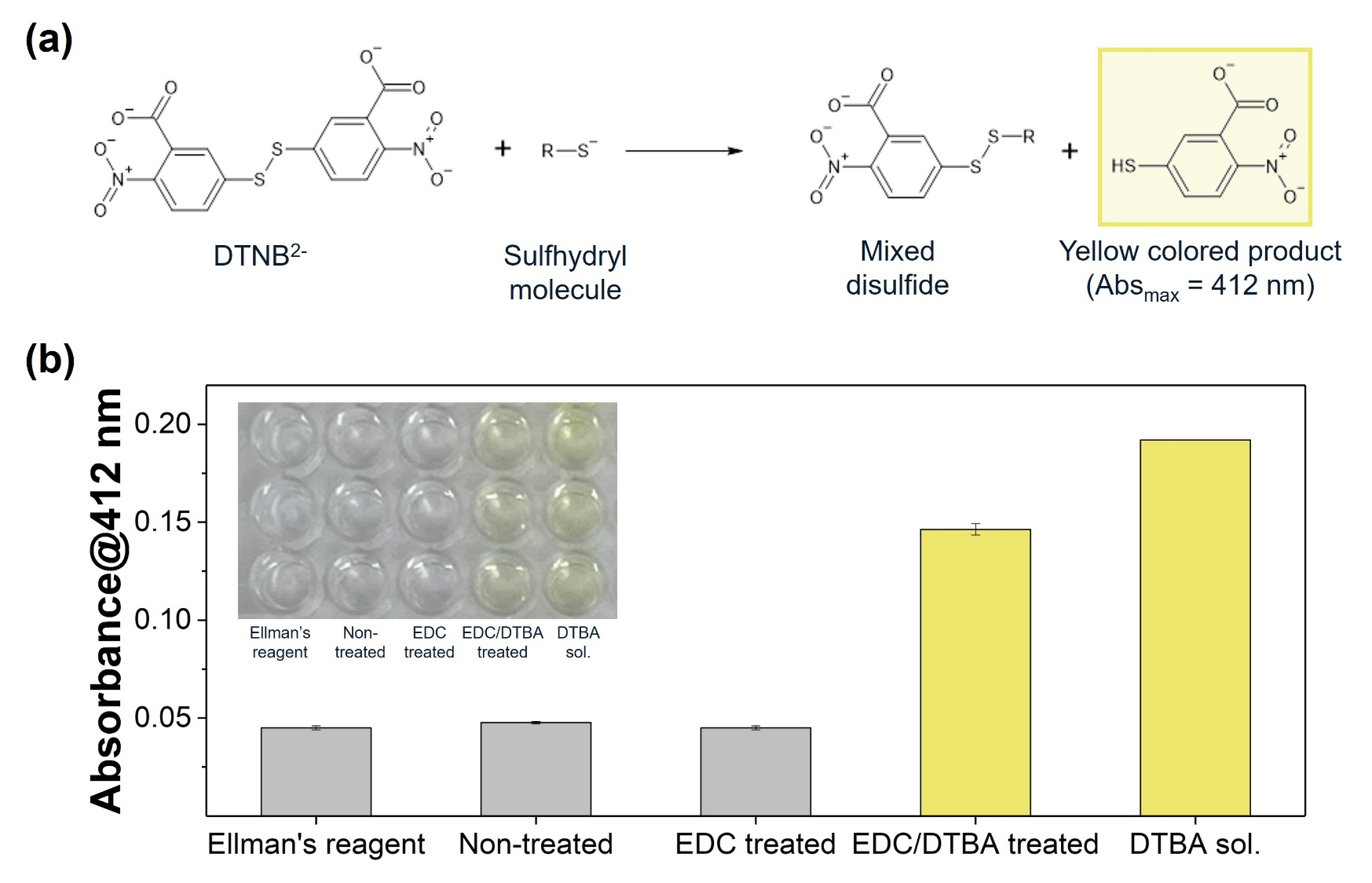
**

**Fig. S2.** Ellman’s test to confirm the functionalization of thiol groups on magnetic beads. (a) The reaction of sulfhydryl groups with DTNB produces a yellow-colored product, 2-nitro-5-thiobenzoic acid, which exhibits an absorbance peak at 412 nm. (b) EDC/DTBA-treated beads showed significant absorbance at 412 nm, comparable to the original DTBA solution.


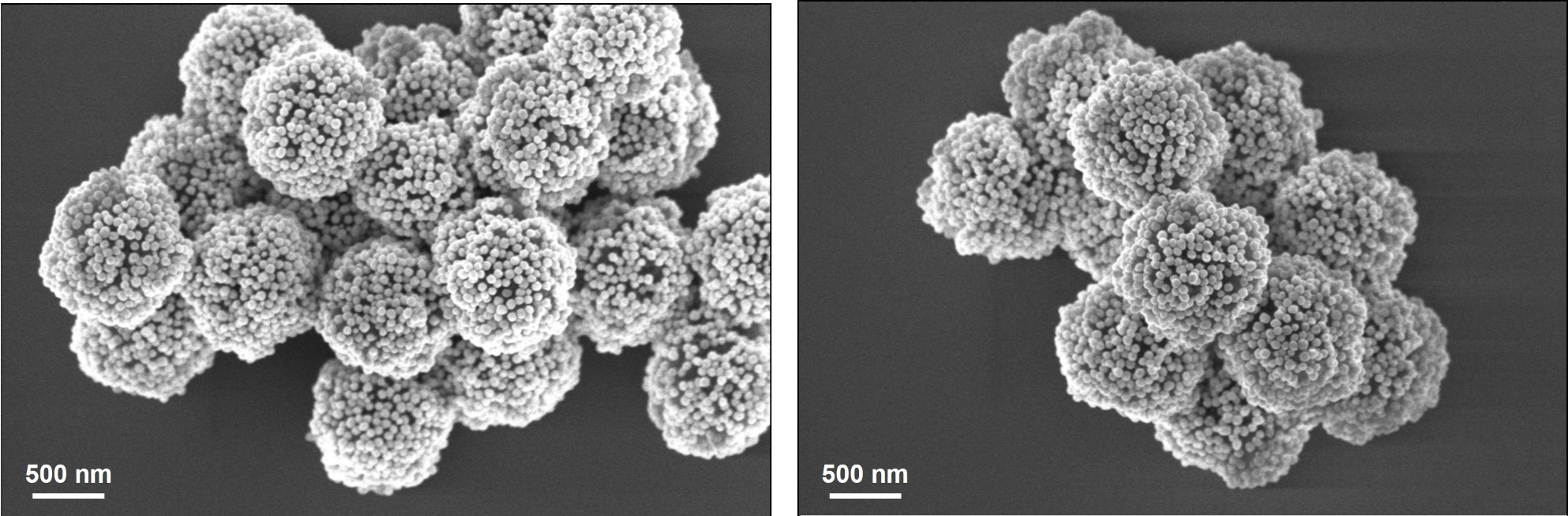


**Fig. S3.** SEM images of magnetic beads conjugated with 70 nm-sized gold nanoparticles.


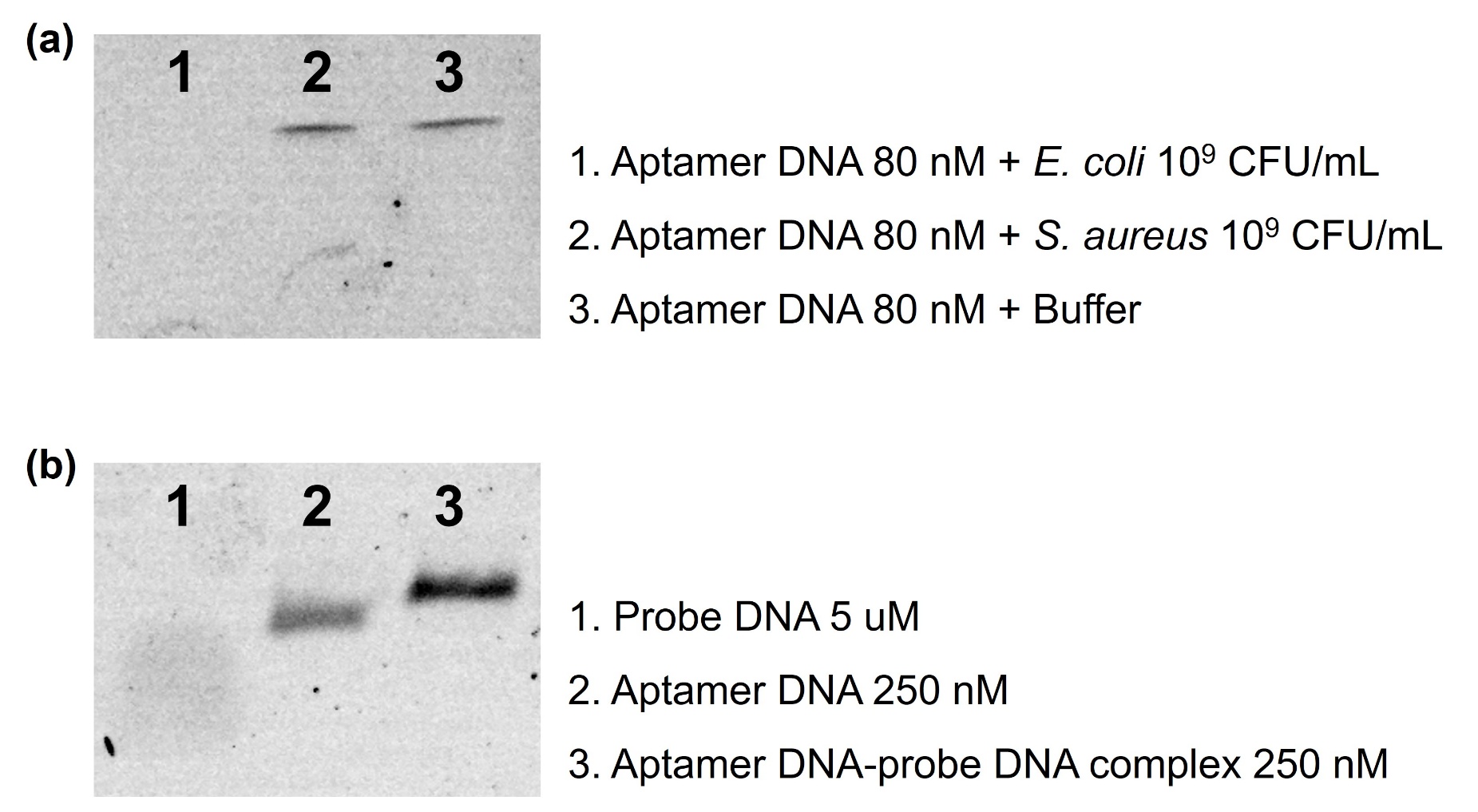


**Fig. S4.** (a) DNA gel electrophoresis results for *E. coli* and *S. aureus*. No bands were observed for the aptamer DNAs that reacted with *E. coli*, indicating specific binding to *E. coli*. In contrast, for *S. aureus*, the electrophoretic band remained visible even after reacting with the aptamer DNAs, suggesting that no interaction occurred between the bacteria and the aptamers. (b) DNA gel electrophoresis results for probe DNA, aptamer DNA, and aptamer DNA-probe DNA complexes. The aptamer DNA-probe DNA complexes migrated more slowly, forming a higher band due to their larger molecular weight compared to the individual aptamer and probe DNA bands.


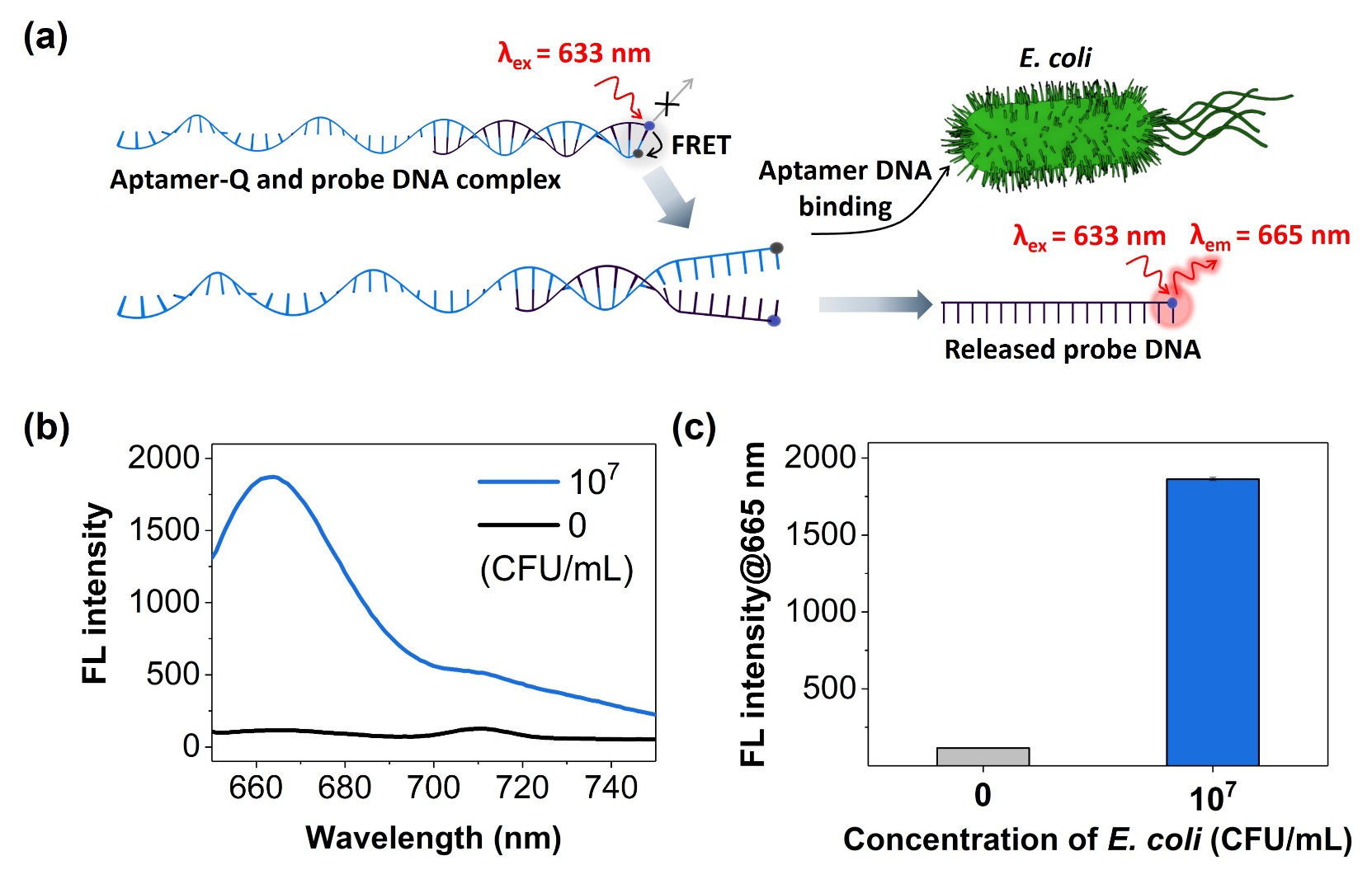


**Fig. S5.** Fluorescence resonance energy transfer (FRET) assays to confirm the release of probe DNAs from aptamer DNA-probe DNA hybridization complexes upon interaction with *E. coli*. (a) A schematic illustration of the assay process, demonstrating how Cy5 fluorescence is restored when the aptamer binds to *E. coli*. (b) Fluorescence spectra and (c) histogram showing fluorescence recovery upon interaction between the aptamer DNA and *E. coli*, confirming that the aptamer DNA's binding to *E. coli* triggered the release of the probe DNA.


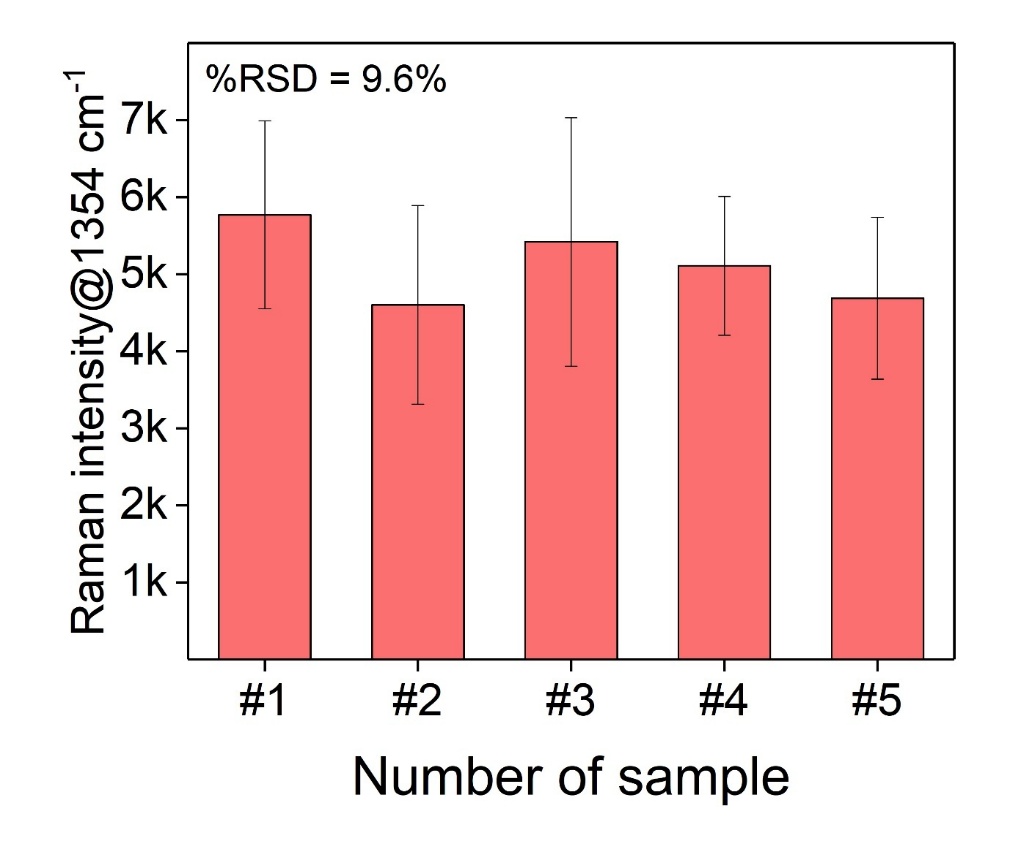

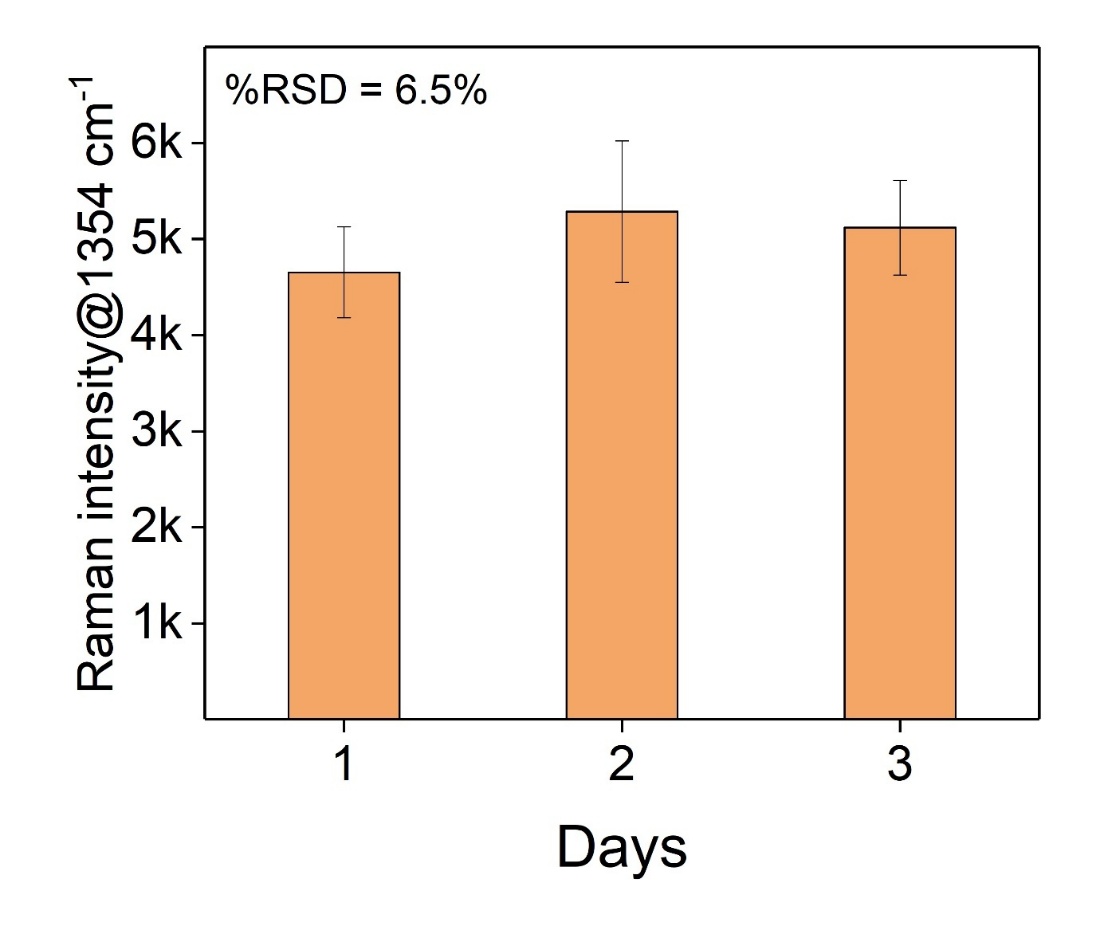


**(a)**

**(b)**

**Fig. S6.** (a) Repeatability test of the Raman signal intensity at 1354 cm^-1^ obtained from the five sample preparations, each at a concentration of 2×10⁵ CFU/mL. Error bars indicate the standard deviation of the triplicate measurements (n=3). (b) Day-to-day reproducibility test of Raman signal intensity at 1354 cm^-1^. Error bars indicate the standard deviation from five replicates (n=5).


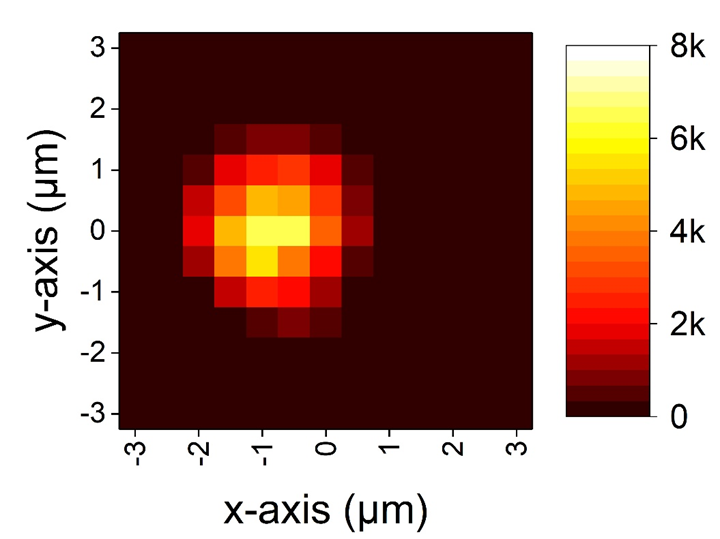

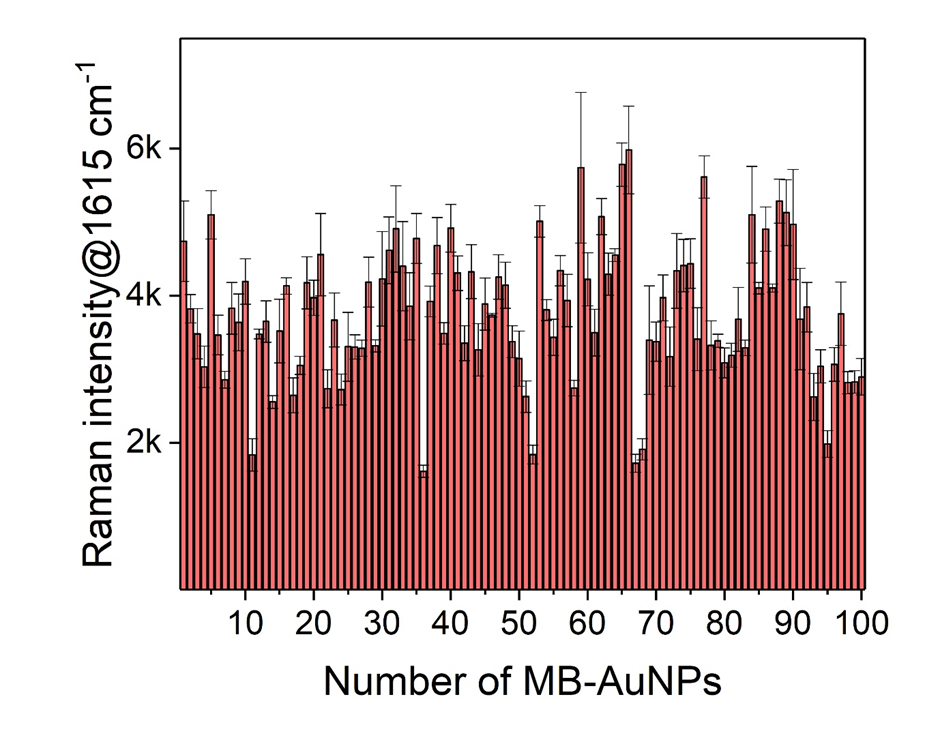


**(a)**

**(b)**

**Fig. S7.** (a) Raman mapping image of a single MB-AuNPs. (b) Variations in the Raman peak intensity at 1615 cm⁻¹ for each particle, obtained from the average Raman spectra data of 100 MB-AuNPs. The error bars represent the standard deviation of three strongest SERS peak intensities within the mapping area of each individual particle. Based on this analysis, the mean Raman signal for the 100 particles was calculated to be 3762.4 ± 919.0, with a %RSD of 24.4%.

**Table S1.** Clinical test results of the SERS-based aptasensor using 21 patient urine samples. Positive/negative (P/N) classification via the SERS-based method was determined using the Raman cut-off value. This value was calculated as Mean + 3σ, where the mean and standard deviation (σ) of Raman intensities at 1354 cm⁻¹ were derived from 10 negative patient samples: 162.37 + (3 × 35.88) = 270.01 (rounded to 270.02 in the dataset).

| Sample name | Urine culture (CFU/mL) |  | SERS-based aptasensor | |  |
| --- | --- | --- | --- | --- | --- |
|  |  | Raman intensity | | P/N | |
| P1 | 1.4 x 10^6^ | 1035 | | P | |
| P2 | 1.4 x 10^6^ | 1174 | | P | |
| P3 | 3.1 x 10^4^ | 391.6 | | P | |
| P4 | 1.6 x 10^6^ | 947.8 | | P | |
| P5 | 1.6 x 10^6^ | 1201 | | P | |
| P6 | 1.5 x 10^5^ | 151.3 | | N | |
| P7 | 3 x 10^5^ | 737.7 | | P | |
| P8 | 1.0 x 10^6^ | 312.2 | | P | |
| P9 | 2.9 x 10^5^ | 756.5 | | P | |
| P10 | 9 x 10^5^ | 450.4 | | P | |
| P11 | 4.5 x 10^5^ | 751.7 | | P | |
| N1 | No growth | 115.2 | | N | |
| N2 | No growth | 147.2 | | N | |
| N3 | No growth | 189.1 | | N | |
| N4 | No growth | 115.0 | | N | |
| N5 | No growth | 138.5 | | N | |
| N6 | No growth | 210.0 | | N | |
| N7 | No growth | 172.5 | | N | |
| N8 | No growth | 188.8 | | N | |
| N9 | No growth | 207.4 | | N | |
| N10 | No growth | 140.0 | | N | |

**Table S2.** Clinical performance evaluation of the SERS-based aptasensor using a confusion matrix.

|  |  | SERS-based aptasensor | |  |
| --- | --- | --- | --- | --- |
|  |  | Positive | Negative | |
| Urine culture | Positive | True positive (10) | False negative (1) | |
|  | Negative | False positive (0) | True negative (10) | |

$$Sensitivity=\frac{True postivie}{True positive + False positive}=100\%$$

$$Specificity=\frac{True negative}{True negative + False positive}=90.9\%$$

$$Accuracy=\frac{True positive + True negative}{Total number of sample}=95.2\%$$

$$Precision=\frac{True positive}{True positive + False positive}=100\%$$

**Table S3.** Comparison of sensor performance in previous studies for *E. coli* detection in UTI diagnosis.

| Detection platform | Detection method | Turnaround time | Assay steps | Limit of detection | Reference |
| --- | --- | --- | --- | --- | --- |
| Immunoluminescence assay | Luminescence | ~40 min | 2 | 2.3x10^3^ CFU/mL | [1] |
| Paper-based analytical device | Colorimetry | ~ 6 h | 1 | 1.57 x 10^4^ CFU/mL | [2] |
| Lateral flow immunoassay | SERS | ~45 min | 2 | 10^2^ CFU/mL | [3] |
| RT-PCR | Fluorescence | ~4 h | 2 | 10 copies/reaction | [4] |
| SERS-based aptasensor | SERS | ~6 h | 1 | 5.9x10^3^ CFU/mL | This study |

References

1. H. Liu, Z. Li, R. Shen, Z. Li, Y. Yang, Q. Yuan, Point-of-care pathogen testing using photonic crystals and machine vision for diagnosis of urinary tract infections, Nano Lett. 21, 2854 (2021). <https://doi.org/10.1021/acs.nanolett.0c04942>
2. J. Noiphung, W. Laiwattanapaisal, Multifunctional paper-based analytical device for in situ cultivation and screening of *Escherichia coli* infections, Sci. Rep. 9, 1555 (2019). <https://doi.org/10.1038/s41598-018-38159-1>
3. P. Wu, W. Zuo, Y. Wang, Q. Yuan, J. Yang, X. Liu, H. Jiang, J. Dai, F. Xue, Y. Ju, Multimodal capture antibody-independent lateral flow immunoassay based on AuNF PMBA for point-of-care diagnosis of bacterial urinary tract infections, Chem. Eng. J. 451, 139021 (2023). <https://doi.org/10.1016/j.cej.2022.139021>
4. N. Hinata, T. Shirakawa, H. Okada, K. Shigemura, S. Kamidono, A. Gotoh, Quantitative detection of Escherichia coli from urine of patients with bacteriuria by real-time PCR, Mol. Diagn. 8, 179 (2004). <http://doi.org/10.1007/BF03260062>
